# Impact of Chinese Herbal Medicine on American Society and Health Care System: Perspective and Concern

**DOI:** 10.1155/2014/251891

**Published:** 2014-02-27

**Authors:** Winston I. Lu, Dominic P. Lu

**Affiliations:** ^1^Savon Pharmacy, El Paso, TX, USA; ^2^Foundation Hospital, El Paso, TX, USA; ^3^University of Pennsylvania, Philadelphia, PA, USA

## Abstract

Many Americans, not completely satisfied with traditional western medicine, have turned to alternative and complementary medicine which explains the increasing popularity of the herbal products and the Chinese herbal medicine. The lack of government regulations and the increasing advertisements by the manufactures have created an impression to the common public that the natural herbal remedies are inherently safer and cheaper than conventional medicine. The skyrocketing rise of healthcare cost and the adverse reaction and side effects incurred from the prescribed drugs have both reinforced such an impression. Herbs in the USA and in many European countries have been prepared as capsules, tablets, teas, lozenges, juice extracts, tincture, and ointments. Most of the herbs are administered as a single herb in the USA and Europe. However, the traditional Chinese herbal medicine contains multiple active ingredients from various herbs and is prepared as concoctions by simmering them for hours to produce pharma-therapeutic properties useful for the treatment of a particular disease. Those prepared concoctions are taken gingerly with specific treatment purposes. In the USA and some European counties, herbs are distributed and labeled as dietary supplements and are taken by many individuals for a long period of time creating some medical and dental complex problems among them, especially in terms of anesthesia-surgery complications. This paper provides insight into basic differences in how herbs are prepared before administration to the patients in China versus a single unprepared herb sold in the USA and Europe. Also addressed are the interdisciplinary issues with health professionals, the proper regulations for better quality control of imported herbs, and the proper warning on the labels of the herbs.

## 1. Impact of Chinese Herbal Medicine in the United States

The World Health Organization estimates that 80% of the world population uses herbal medicine [1]. Many Americans, not satisfied with western medicine, have turned to alternative and complementary medicine resulting in increasing popularity of herbal products and traditional Chinese medicine, especially acupuncture and Chinese herbal medicine. Traditional Chinese medicine (TCM) includes 5 branches, namely, acupuncture and Moxibustion, Chinese herbology (commonly known as Chinese herbal medicine), Qigong healing, Tuina therapeutic massage, and Chinese dietary therapy. This paper will focus on the impact of Chinese herbology (Chinese herbal medicine) on the American society and the health care system.

In America and Europe, traditional folk medicine uses many herbs as in Chinese herbal medicine (CHM), but they are often handled and prepared differently with different therapeutic results. There are very few sources of information in the USA about CHM, although many American herbs have the similar properties as their Chinese counterparts. While herbs are considered drugs in China, herbs are consumed in the USA as nutritional or food supplements without necessary warning labels on containers. We provide a background of CHM and information on herbal preparation.

## 2. Background Information

Generally speaking, traditional Chinese medicine offers an alternative to the potentially toxic effects of western medication, since acupuncture does not inject any drug into the body and many Chinese herbs are natural products, tend to be milder, and can be combined with western medicines. For example, Chinese herbal medicines improve and augment the effectiveness of western medications in the treatment of liver cirrhosis, arthritis, and cancer, thereby decreasing the dosage and adverse side effects of western chemotherapies [2–4]. Since 1974, when former president Nixon's third exchange delegation to China covered the subject of herbal pharmacology, much information had been shared by eastern and western scientists. Western research into CHM has appealed to both Chinese and western medical establishments [5], but concerns remain. One concern is that in China, few regulations have been applied in herb producing areas or plants and some merchants ignore existing regulations, resulting in quality control problems [6].

Since herbs are widely available in pharmacies throughout the USA, many people assume pharmacists are aware of drug-herb interactions and appropriate warnings, but this is not true. There is also the problem of improper self-administration of herbs by patients who do not inform their physicians, dentists, or pharmacists and many American clinicians lack sufficient knowledge of Chinese herbs to advise patients [7, 8].

## 3. Current Status of Chinese Herbal Medicine in American Society

Because therapeutic effects of Chinese herbs have been exaggerated or distorted, herbs have often been misused. For example, ma huang has been used in China for several thousand years as an antiasthmatic medication and researchers subsequently extracted from it the alkaloid ephedrine. In the USA, ephedrine has been illegally processed into a stimulant “designer drug” (ecstasy) with ma huang as a major ingredient. Hospitalization and deaths have occurred after mixing ecstasy with liquor, heroin, and sometimes cocaine [5].

To avoid government regulation many American manufacturers and distributors have no cautions or warnings on labels. For example, Ginseng (Panax Chinese or Korean) has a good therapeutic effect with cardiovascular benefits especially for convalescent patients, and inadequate warnings have allowed indiscriminative usage and incidents of Ginseng Abuse Syndrome (GAS). GAS is manifested by diarrhea, skin lesions, CNS stimulation, and interference with homeostasis. Ginseng also potentiates the antiplatelet effect of Coumadin, aspirin, and NSAIDs (although okay with COX-2 sedative) and taking these drugs with ginseng may pose surgical risks. Siberian ginseng may elevate the blood pressure and enhance irritability with long term use, although it is known to enhance t-lymphocyte activity and may improve antibiotic effectiveness [7].

*Ginkgo biloba* often combined with Herba Ephedrae (mahuang), Pruni Armeniacae, and others for cough and wheezing. This herb alone is known in China to be slightly toxic and not to be taken in large quantity or long term. But no such warning is labeled in the product information to the consumers in the USA. It is known to decrease platelet aggregation with the anticoagulant warfarin which could interfere with homeostasis. *Ginkgo biloba* will also react with narcotic analgesics causing hypotension [7, 8]. Dong quai (angelicae sinensis) [6], used in China for pain and trauma or “female problems,” can also interfere with warfarin in high doses. It may potentiate skin cancer or raise blood glucose levels. People who are diabetic, have heart problems, or are taking anticoagulants should not take dong quai. Another popular herb known as licorice root (*Glycyrrhiza glabra*) and its Chinese counterpart (*Glycyrrhiza uralensis*) contain salts of glycyrrhizic acid. This glycoside can intensify platelet aggregation thereby decreasing coumadin function [9]. In China, it is common knowledge that herbs cannot be taken continuously and certain foods must not be taken with certain herbs. From time and time, doctors of traditional Chinese medicine must be consulted for proper herbal dosage adjustment, to meet individual needs and provide necessary warnings. These precautions are not followed in the USA.

## 4. Chinese Method of Decoction Preparation

One main difference between western herbal medicine and CHM is that in the USA herbs are sold by having the herb ground into powder and encapsulated or by putting together the powder of several herbs into one capsule. But in CHM, herbs are often combined under certain preparation and treatment. CHM, through thousands of years of experimentation and clinical testing, had developed sophisticated and complex methods of herbal preparation. Their therapeutic effects are often achieved through the synergistic and combined actions of different compounds. In addition, certain minerals and animal parts are sometimes used in these concoctions. Organic compounds of high complexity tend to decompose to lower weight compounds upon exposure to heat (during boiling preparations of herbs) and these compounds may react with one another to form other compounds. Molecular properties such as water solubility, molecular affinity between compounds, and the length and type of molecular bonding play a major role in how they react. Under such complex pharmacodynamic processes with various decompositions, reactions, and preparative conditions, it is likely that the end products may have entirely different pharmacologic properties than did the original herbal ingredients. However, under FDA labeling guidelines, original ingredients are listed and this may confuse consumers and pharmacists [2].

Throughout the history of CHM, there have been different ways in which medicinal substances can be combined. Combination classifications are based upon disease pattern symptom complex, etiology, form of application (e.g., external applied or internally ingested), type of usage (gynecology, pediatrics, ophthalmology, etc.), organ, treatment strategy, and composites of the above. The art of the combining is by putting together two or more medicinal substances so as to promote therapeutic effectiveness, to minimize toxicity or side effects, to accommodate complex clinical situations, and to alter the actions of the substances. Chinese herb medicines are usually prepared as decoctions (tang, literally soups) the most common form in which CHM is taken in China, but in the USA there is resistance to using decoctions.

Traditionally, certain combinations of herbs were avoided because they either reduce each other's effectiveness or lead to toxicity or undesirable side effects. There are reported cases of mutual antagonism and cases of mutual incompatibility in the Chinese Classic of the Materia Medica. For example, Radix Ginseng antagonizes the herb Excrementum Trogopterori seu Pteromi which is used for gynecological disorders with energy deficiency and blood stasis. Also cortex cinnamomi cassine (cinnamon bark) antagonize halloysitum rubrum (kaolin) that is used for chronic dysenteric diarrhea, whereas radix glycyrrhizae uralensis (licorice root) is compatible with radix euphorbiae sue knoxie (Japanese thistle) and seaweed. There are also certain foods to be avoided by patients taking certain medical substances and, in general, patients taking herbs should avoid cold, greasy, or other hard to digest foods [10].

## 5. Methods of Herbal Detoxification

Since the early nineteenth century, scientific research has attempted to understand the actions and properties of CHM herbal substances. It was also during this time that most modern drugs were developed. Many herbs sold in the USA are harvested and ground from the original plant form without any TCM processing or preparation. Without the processing to remove toxicity or undesirable side effects, such products could be toxic. In CHM, there are several processing methods to achieve the desired medicinal substances from herbal plants. For example, using alcohol to process *Angelicae Sinensis* to extract its volatile oils and treating Rhizoma Corydalis Yanhusuo with salt allow its alkaloids to dissolve in water. Radix Rehmanniae Glutinosae (sheng di huang, the dried form of the herb) is cooling in nature and can be cooked in wine and then dried to become Radix Rehmanniae Glutinosae Conquitae for usage as a tonic. Other methods to prepare herbs include dry frying, browning, frying with honey or vinegar, baking, roasting, steaming, and boiling with water. Heating and boiling frequently denature the toxic proteinous parts of the herbs [10].

## 6. Discussion

In the last 50 years, tremendous amount of acupuncture research has been done in Europe, Asia, and the United States to unlock the mysteries of acupuncture. Those researches have helped the modern medicine to know more about how human body works. Whereas acupuncture is well received by both American medical establishments and the public alike, the attitude to herbal medicine is entirely a different matter.

The increased use of herbs as supplements is problematic as consumers often do not have understanding of the therapeutic effects of these herbs. In fact, some herbal supplement use is prevalent in the facial/cosmetic surgery population, especially in older female patients. Some herbs cause coagulopathy, hypertension, or dry eyes. Abstaining from herb usage two weeks before surgery is recommended; nearly half of patients reported taking herbs, which cause intraoperative bleeding [11]. In addition, in China, many of these herbs were not traditionally meant to be used therapeutically as a single unprepared product, and it is questionable whether they share the same effect as the preparations used in CHM concoction by mouth. Now the unconventional intravenous injection of herbal preparations bypassing the gastrointestinal tract has caused some allergic reaction in some patients, which is relatively uncommon if the properly prepared herbs are taken by mouth. In fact, a total of 109 varieties of Chinese medicine injections have been approved by the State Food and Drug Administration of China, all of which have the potential to induce adverse drug reactions that include systemic anaphylaxis, anaphylactic shock, acute intravascular hemolysis, hepatorenal damage, skin lesions, cardiac damage, respiratory distress, and GI disorders [12]. Furthermore, the tendency for consumers to use multiple unprepared herbs in conjunction with one another and in addition to prescription medications is problematic as most consumers and, in fact, most physicians and pharmacists are unaware of their potential drug interactions. These single unprepared products, if taken without professional monitoring, can pose potential risks to patients and those products can cause interdrug reactions with the prescribed medications the patients are taking (see Table 1). The lack of information concerning these cross-interactions and a general lack in training are a potentially significant health issue. This issue is perpetuated by the listing of these herbs as supplements, which do not require significant manufacturer quality control and limit the FDA's oversight concerning possible adverse effects and drug interactions between these herbs and various medications.

The status of herbal supplements as dietary supplements with little oversight is designed to increase the availability and accessibility of these supplements to the general public. This has increased the incorrect widespread use of Chinese herbs by mainstream consumers. However, unless proper precautions, studies, and oversight of the herbs are developed there lies the possibility that these drugs may become less utilized when the potential hazards of these herbs become more publicized.

Qualitative and quantitative testing of the active ingredients of traditional herbal preparations is also necessary for the effective utilization of Chinese herbal medicine. Lack of quality control is a major problem as unscrupulous manufacturers of herbal medicine have blended western medicine into CHM to exaggerate its therapeutic effect without labeling it as such. This is because pure CHM is milder and requires longer time to take effect than western medicines, which reach peak therapeutic effect faster but with more side effects than CHM. For example, acetylsalicylic acid has been added to antipyretic herbals and Butazolidin was added to arthritic herbals to boost their effect.

Herbal doping by athletes to improve performance is less of a problem in the United States than other countries due to stringent testing. Nevertheless, doping usually involves ginseng and in some cases musk pod from the musk deer. Ginseng is used as an adaptogenic throughout the world. In the United States, the use of ginseng is skeptically viewed as an athletic performance enhancer due to studies that have shown varying benefits in fatigued athletes. These results may reflect that some commercially available ginseng products contain little to no active ingredient. Some ginseng products have even been shown to contain added anabolic steroids [13, 14].

The use of and demand for herbs as supplements continue to rise and when used as isolated products, it is questionable whether they have the same effects as CHM concoctions. The authors remain hopeful about the future of combining western and eastern medicines to attain their full potential for the benefit of patients. Regulatory agencies in the United States and China will need to cooperate. Continuing education courses and dialogue should be developed for pharmacists, physicians, dentists, and practitioners of traditional Chinese medicine to keep up with current research. Herbal medicine and drug interactions need to be thoroughly understood in order to safeguard public safety and health [15–17].

To address the issues of inadequate knowledge base and understanding of herbal medicine by health professionals, we recommend increased exposure to herbal medicine in the curriculums of medical, dental, pharmacy, and nursing schools. Increased sponsorship of clinical studies to study and address the comparative and acceptable uses of herbals would better clarify their safe and effective use in an objective format more easily accepted and understood by health care professionals. For qualified and interested health professionals, postcertification programs might be developed to unify fields of study, such as a dietary certificate with an emphasis on supplemental nutrition and herbal medicine.

Herbs are not patentable and knowledge acquired through research would soon become public domain. It is desirable that either a special division of the FDA or a separate regulating agency be created to regulate herbs (both CHM herbs and traditional herbs) used in the United States.

## 7. Perspectives and Conclusion

Herbal medicine and western medicine may be seen as totally separate distinctive entities and disciplines, but, in fact, both can be combined together for better results. They can be quite complementary to each other for synergizing the therapeutic effects. With increasing public awareness of herbal medicine, it is the hope of the authors that complementary and alternative medicines can be added to mainstream schools of health sciences by developing a view of the human body that includes oriental concepts. In addition, a public health campaign can be launched to educate about the merits and hazards of indiscriminate use of herbs.

## Figures and Tables

**Table 1:** Some common unprepared Chinese herbs in the USA and their potential risks and interdrug reactions when taken without professional monitoring.

Herbal names	Botanic names	Medical uses	Potential risks
Aloe (other names: hsiang-dan, lu-hui)	*Aloe barbadensis*/*capensis*/*vera *	Depresses the action potential generation and conduction at neuromuscular junction processes, analgesic, and anti-inflammatory effects. Increases the collagen content of granulation. Tissue contributed to wound healing, sometimes used to treat AIDS, diabetes, asthma, stomach ulcers, immune weakness, evacuation relief, anal fissures after rectoanal surgery, fungal diseases, constipation, colic, and worm infestations.	Loss of electrolytes, potassium, this hypokalemic effect enhanced in conjunction with thiazide diuretics, loop diuretics, licorice, and corticosteroids (increase the action of cardiac glycosides and antiarrhythmic drugs).

Huang-Qi (other names: superior Chinese astragalus)	*Astragalus* species	Used for respiratory infections, immune depression, cancer, heart failure, viral infections, liver disease, and kidney disease. Hyperthyroidism, hypertension, insomnia, diabetes, genital herpes, AIDS, and the side effects of chemotherapy.	Bleeding when used with other anticoagulant, antiplatelet, or antithrombotic agents. It is incompatible with opiates.

Chinese rhubarb, da-huang	*Rheum palmatum *	Constipation, appetite stimulant, painful teething, delirium, edema, and diarrhea.	Electrolyte loss (especially potassium leading to hyperaldosteronism and enhanced effects of radioactive drugs). Long term use causes arrhythmias, nephropathies, and bond loss.

Dandelion (other names: lion's tooth, endive)	Taraxacam offinae/laevigatum	Dyspeptic conditions, urinary tract infections, liver and gallbladder, loss of appetite, fluid retention, constipation, rheumatism, and diabetes.	Not to be used with diuretics, antihypertensive agents and oral hypoglycemic, and mammal and lactating related problems.

Ephedra, ma huang (other names: natural ecstasy, fen-phen)	*Ephedra sinica *	Used as CNS stimulant for appetite suppressant, a nasal decongestant, bronchial asthma, joint symptoms, inability to perspire edema, and pain in the bones.	May alter effects of MAO inhibitors, ephedrine, B-blockers, phenothiazines, and Sudafed. Higher dosages result in blood pressure and cardiac rhythm disorders; it has an additive effect with caffeine and decongestants and heart rhythm disturbances when used with halothane.

Garlic (other names: da-suan)	*Allium sativum *	For elevated lipid levels, age-related vascular change and arteriosclerosis, inflammatory respiratory conditions, gastrointestinal ailments, diabetes, constipation, and joint pain.	Decrease in hematocrit values and plasma viscosity; concomitant use with Coumadin antiplatelet drugs such as aspirin and dipyridamole could increase the effect of bleeding. Risk of bleeding increased with ginkgo or high-dose vitamin E and may increase serum insulin levels.

Ginkgo, *Ginkgo biloba *(other names:xGinkgo)		For organic brain dysfunction intermittent claudicating, vertigo and tinnitus, improving concentration, asthma, hypertonic, erectile dysfunction, and angina pectoris.	Spontaneous bleeding due to potent inhibitory effect on platelet-activating factor; care when used with aspirin and other anticoagulant hypertension with thiazide diuretics.

Ginseng (other names: Chinese Red Panax)	*Panaxginseng*/*quinquefolius *	For fatigue and debility, concentration, loss of appetite, cachexia, anxiety, impotence and sterility, neuralgia, and insomnia.	Hypoglycemic effects, hypotension resulting with prolonged high-dose ginseng with caffeine, adverse effects with oral hypoglycemic and MAO inhibitors, concomitant use with aspirin, NSAIDs, heparin, and warfarin should be avoided.

Green tea (other names: Chinese matsu-cha)	*Camellia sinensis *		Vitamin K in green tea interferes with Coumadin, decreases the absorption of alkaline drugs.

Licorice	*Glycyrrhiza glabra *	For cough/bronchitis and gastritis, also used for appendicitis, constipation, increase milk production, micturition, gastric ulcers, headache, sore throat, spleen disorders, dehydration, and chronic fatigue syndrome.	Hypokalemia, hypernatremia, edema, hypertension, and cardiac complaints. Additive effect with furosemide and thiazide diuretics. Hypokalemic effects potentiate digitalis toxicity. Severe ventricular tachycardia of the torsade de pointes type resulted with the concomitant use of antiarrhythmic agents and may prolong the half-life of cortical increasing its effectiveness and its side effects.

Papaya	*Carica papaya *	For gastrointestinal digestive complaints, athletic injuries, and herniated vertebral disks.	Fibrinolytic effect, tendency to bleed due to interaction with warfarin and increased INR levels.

Prickly ash (other names: toothache tree, stanberry)	*Zanthoxylum americanum *	For toothache, intestinal gas, to promote circulation, and rheumatism.	Promote bleeding when used with aspirin or other blood thinners.

Turmeric (other names: Jiang huang)	*Curcuma longa *	Dyspeptic complaints and loss of appetite, also cancer, gallstones, intestinal gas osteoarthritis, and rheumatoid arthritis.	Alter the action of Coumadin, NSAIDs, and immune system suppressants.

Wild Yam (other names: China root)	*Dioscorea villosa *	For rheumatic conditions, gall bladder colic, dysmenorrheal, and cramps.	Decrease the anti-inflammatory effect of indomethacin, additive effect with estrogen.
